# Supplementary material for: Single serine on TSC2 exerts biased control over mTORC1 activation mediated by ERK1/2 but not Akt
Source: Life Sci Alliance. 2022 Mar 14;5(6):e202101169. doi: 10.26508/lsa.202101169 (PMC8921838; doi:10.26508/lsa.202101169)
Supplement: Supplementary file 1 [file LSA-2021-01169_SdataF1.1.pdf]

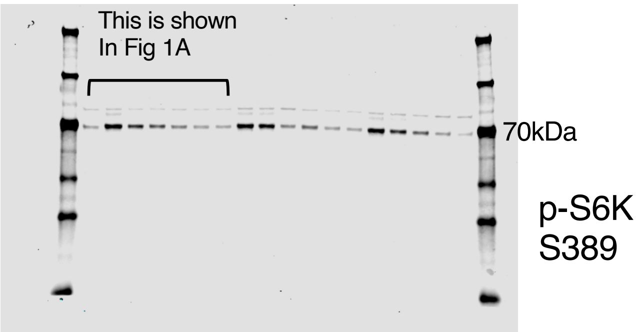

p-S6K
S389

<u>Lanes</u> <u>Condition</u>

1,7,13 - Vehicle
2,8,14 - 100 nM ET1
3,9,15 - ET1+0.01 μM Sch
4,10,16 - ET1+0.1 μM Sch
5,11,17 - ET1+1 μM. Sch
6,12,18 - ET1+10 μM Sch

Lanes 1-6 shown in Figure

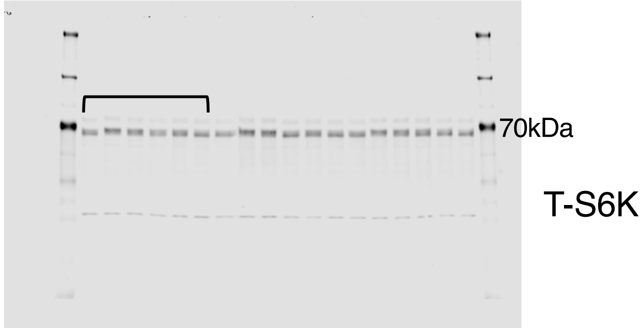

T-S6K

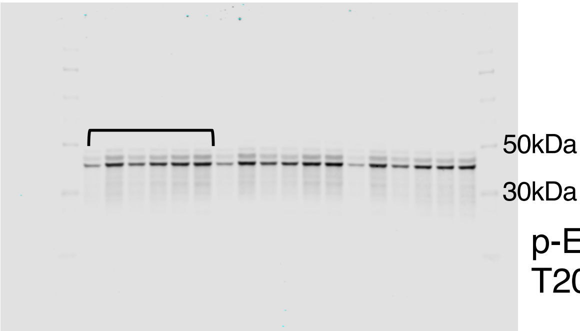

p-ERK1/2
T202/Y204

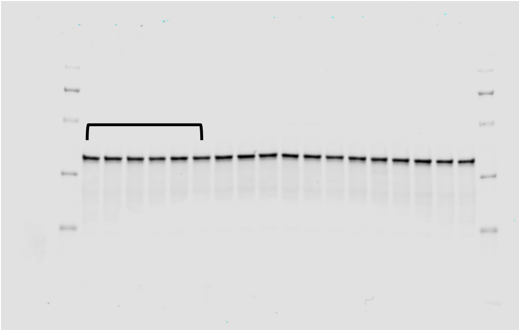

T-Akt

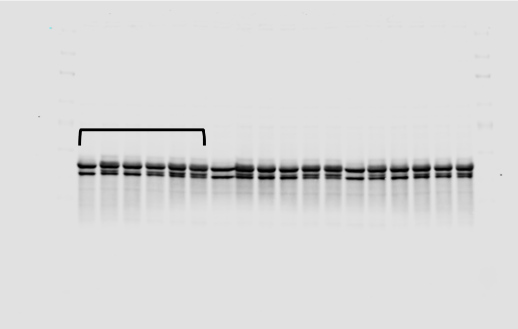

T-ERK

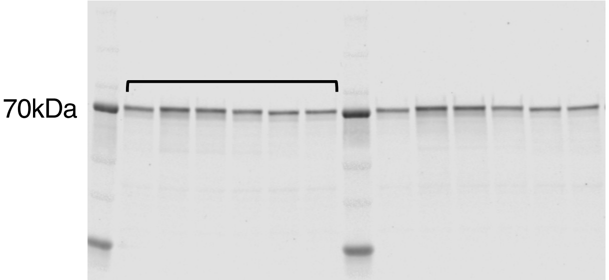

p-RSK2
S227

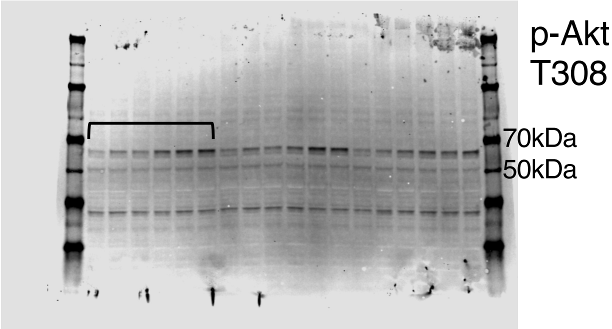

p-Akt
T308

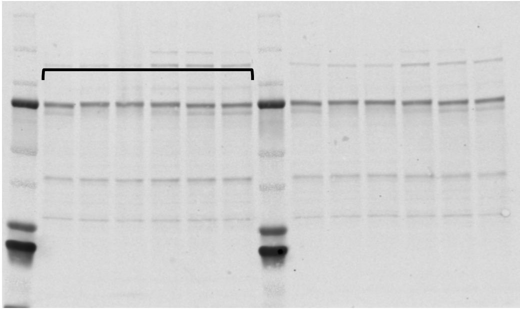

T-RSK2

Figure 1A Raw Gels

Gel has three experimental replicates. In each, there are 6 lanes; lanes 1,2 and 4,5,6 are relevant to the experiment presented. Lane 1 is vehicle, 2 ET-1, and 4-6 ET1 with addition of 0.15, 1.5, or 15 μM MK 2206.

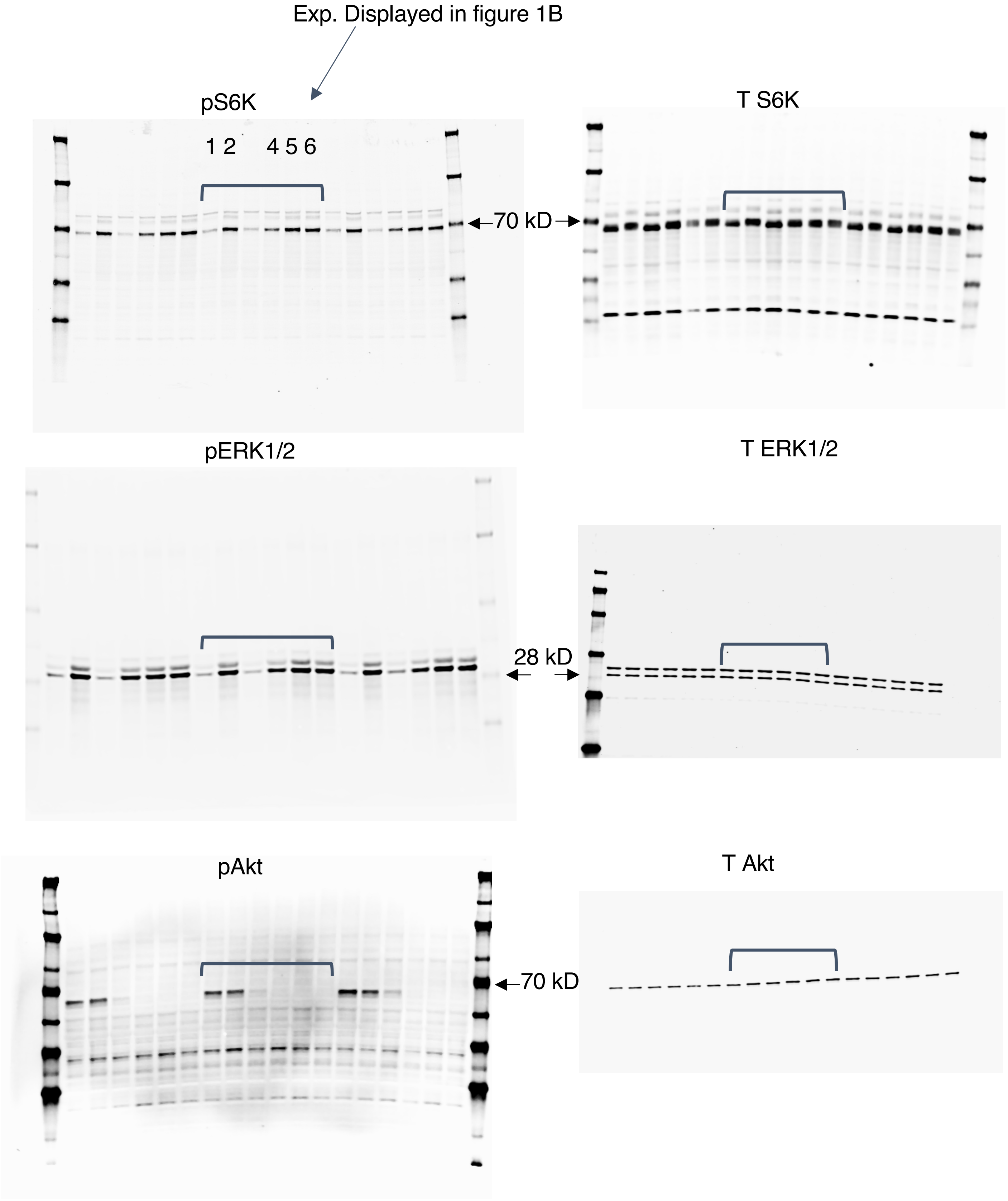

Figure 1B Raw Gels

pS6K

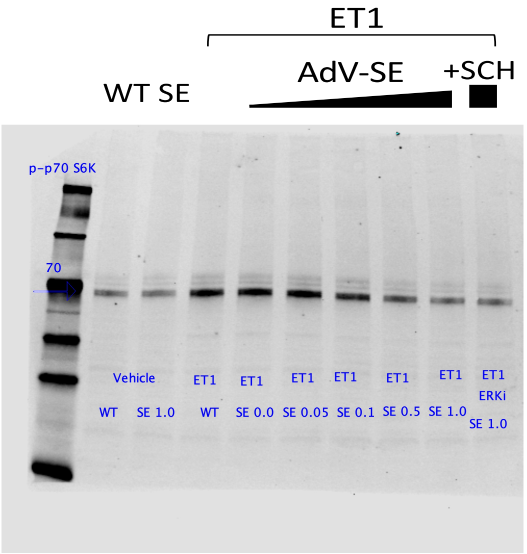

T S6K

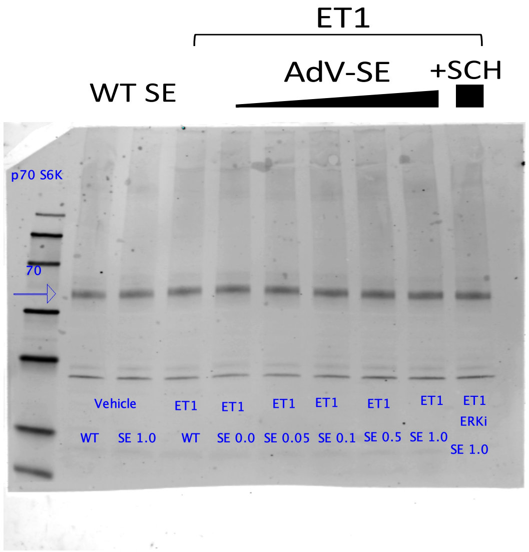

pERK

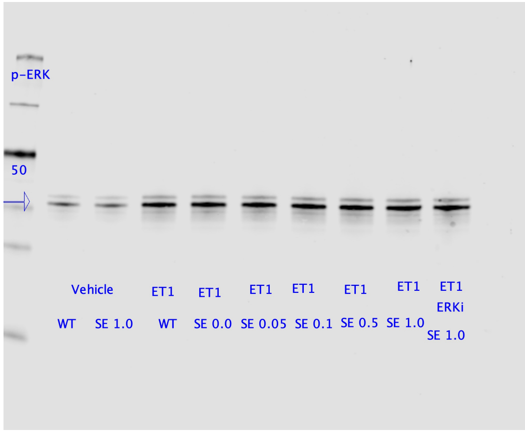

T ERK

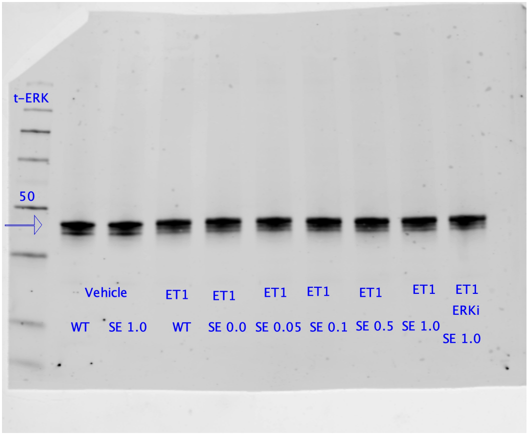

P Akt

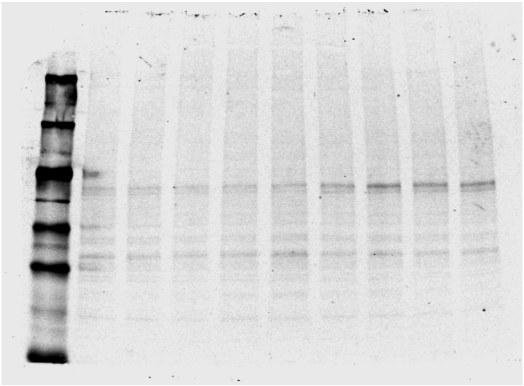

T Akt

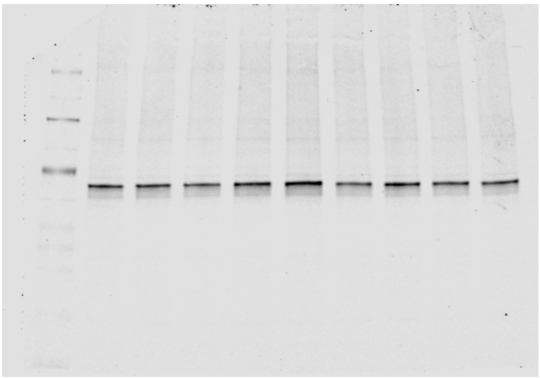

Figure 1C Raw Gels
