# Supplementary material for: Single serine on TSC2 exerts biased control over mTORC1 activation mediated by ERK1/2 but not Akt
Source: Life Sci Alliance. 2022 Mar 14;5(6):e202101169. doi: 10.26508/lsa.202101169 (PMC8921838; doi:10.26508/lsa.202101169)
Supplement: Supplementary file 3 [file LSA-2021-01169_SdataF2.1.pdf]

1 – WT Vehicle
2 – SA vehicle
3– WT + ET1
4 – SA + ET1
5 – WT + ET1 + Sch
6 - SA + ET1 + Sch
7 – WT + ET1 + Rapamycin
8 - SA + ET1 + Rapamycin
9-16 repeats sequence

pS6K

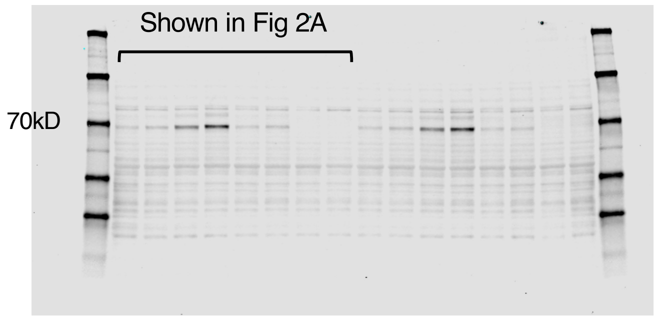

T-S6K

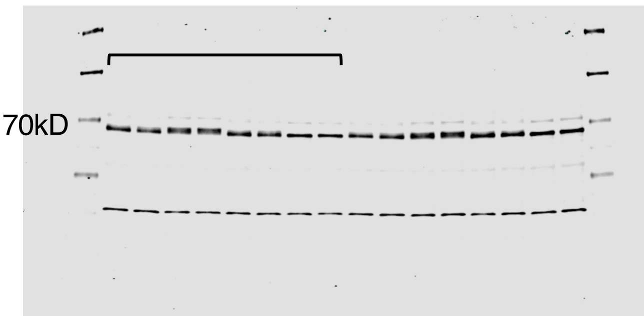

pERK

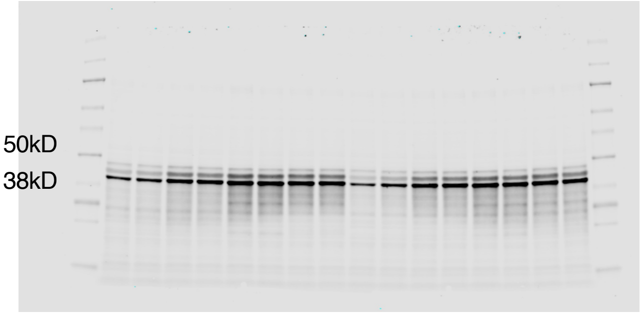

T-ERK

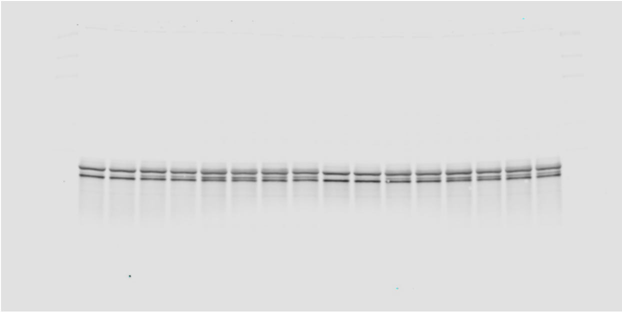

TSC2

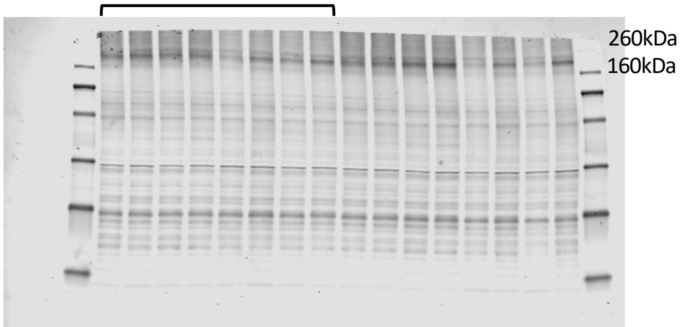

Figure 2A

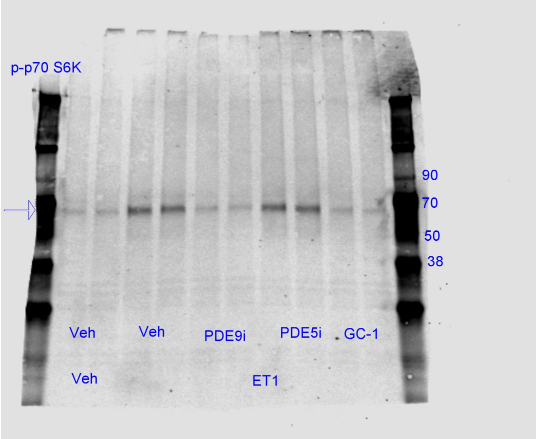

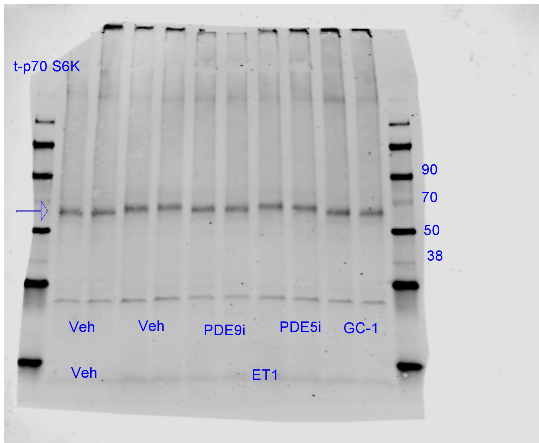

Total Protein – gels 1-2

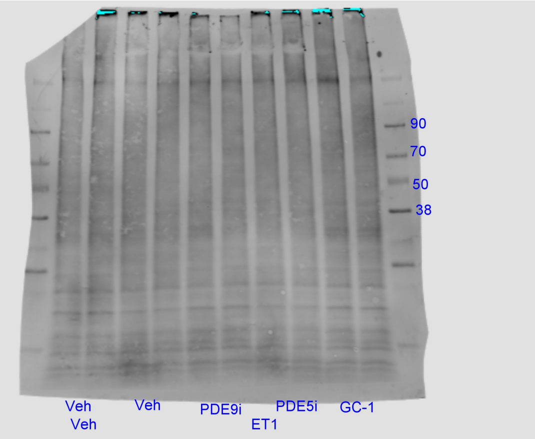

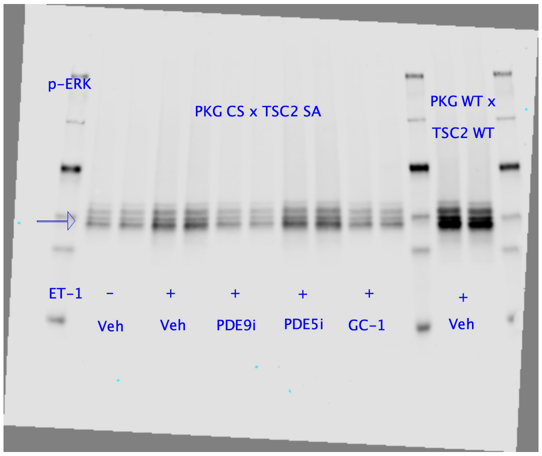

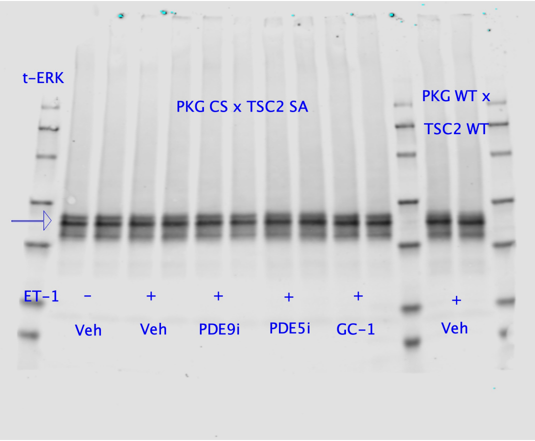

Total Protein – gels 3,4

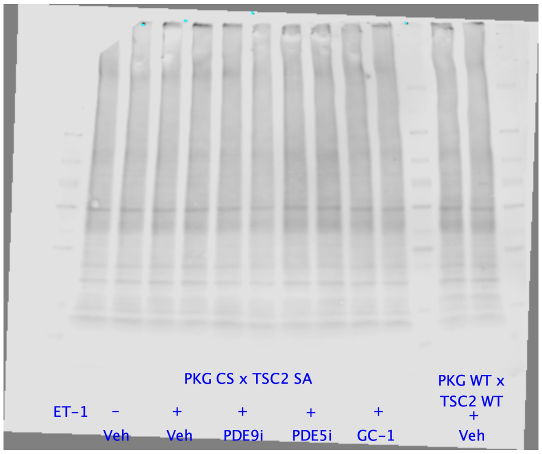

Figure 2C
