# Supplementary material for: Single serine on TSC2 exerts biased control over mTORC1 activation mediated by ERK1/2 but not Akt
Source: Life Sci Alliance. 2022 Mar 14;5(6):e202101169. doi: 10.26508/lsa.202101169 (PMC8921838; doi:10.26508/lsa.202101169)
Supplement: Supplementary file 5 [file LSA-2021-01169_SdataF3.1.pdf]

6- replicates of same experiment. Control; insulin; insulin + Sch (ERK inhibitor); insulin + MK (Akt inhibitor)

Data shown in Figure 3A is from the 5th experiment in the series.

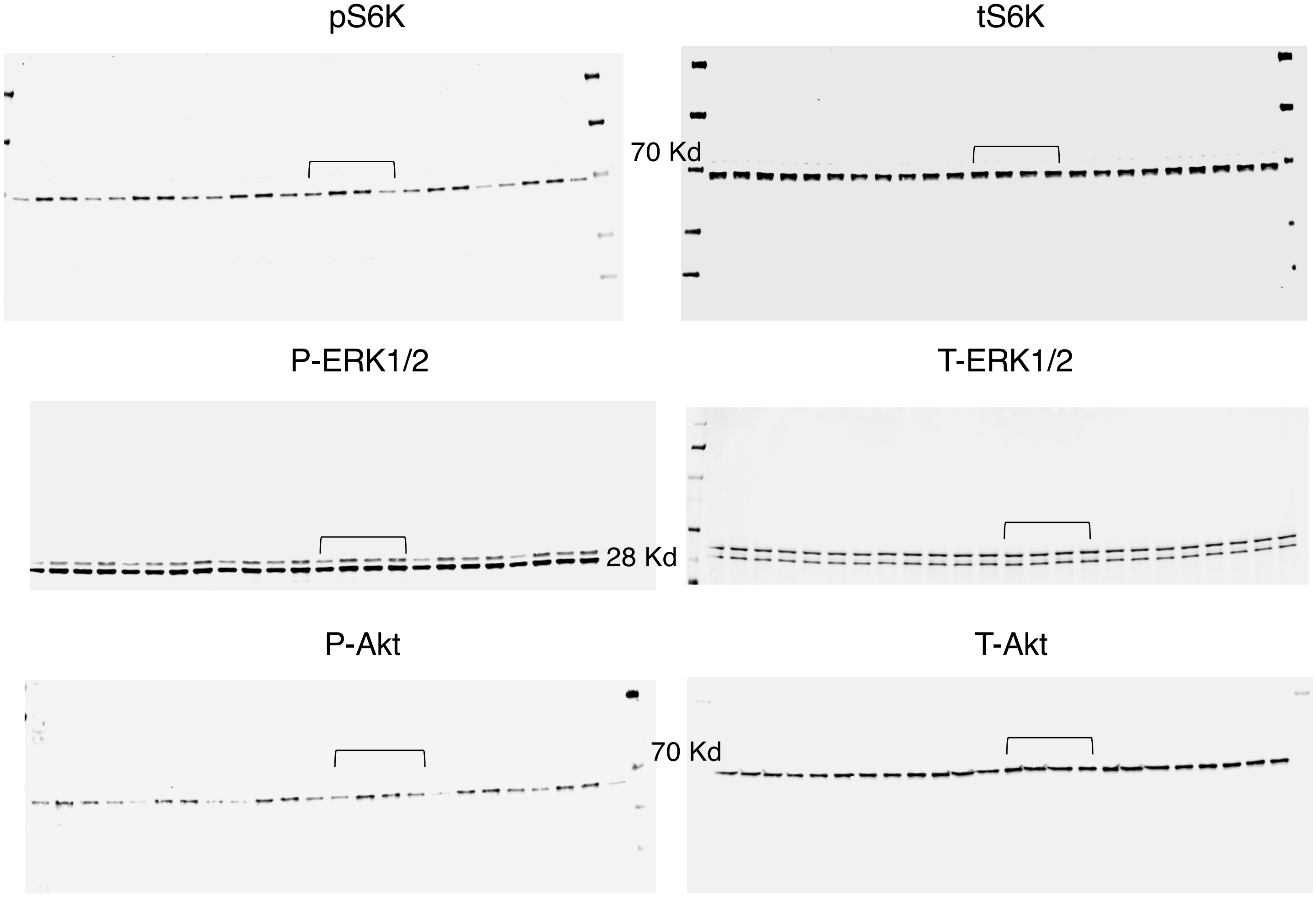

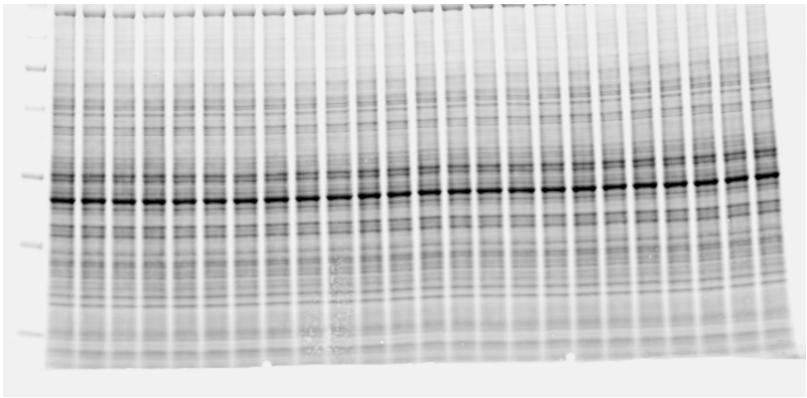

Figure 3A

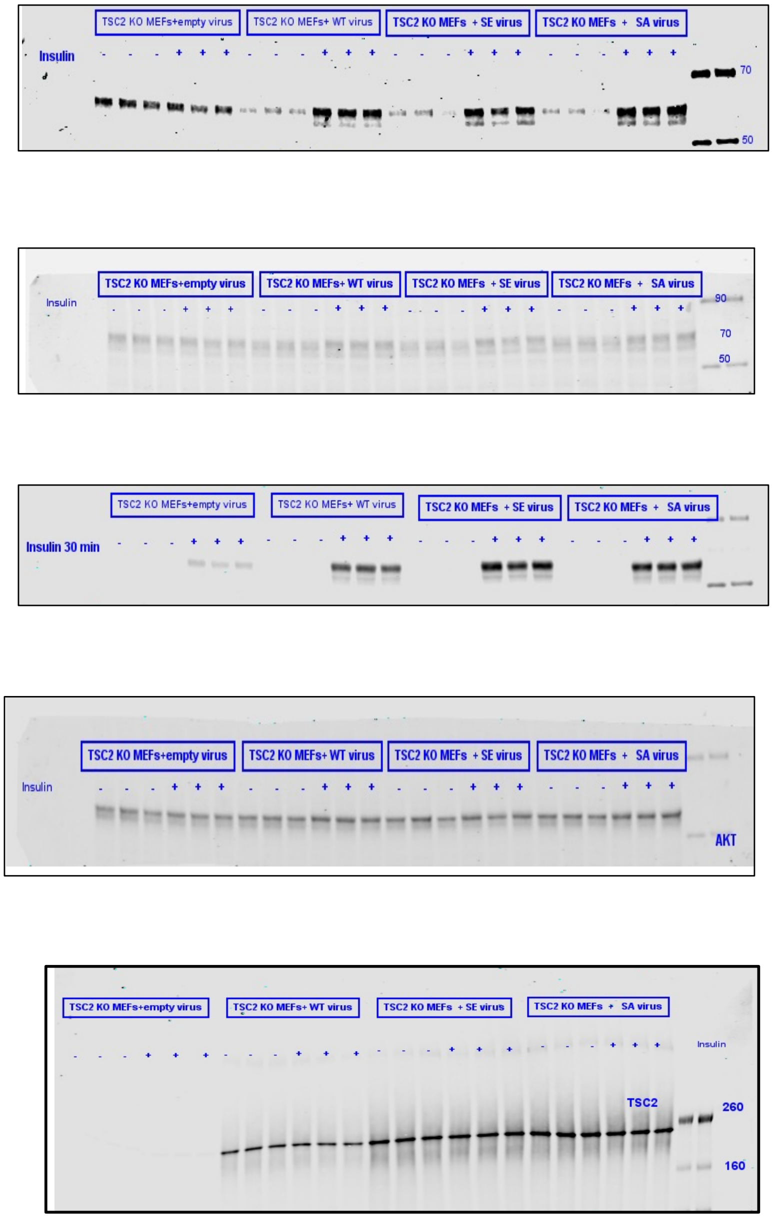
TSC2 KO MEFs+empty virus
TSC2 KO MEFs+ WT virus
TSC2 KO MEFs + SE virus
TSC2 KO MEFs + SA virus
Insulin
- - - + + + - - - + + + - - - + + + - - - + + +
70
50
TSC2 KO MEFs+empty virus
TSC2 KO MEFs+ WT virus
TSC2 KO MEFs + SE virus
TSC2 KO MEFs + SA virus
Insulin
- - - + + + - - - + + + - - - + + + - - - + + +
90
70
50
TSC2 KO MEFs+empty virus
TSC2 KO MEFs+ WT virus
TSC2 KO MEFs + SE virus
TSC2 KO MEFs + SA virus
Insulin 30 min
- - - + + + - - - + + + - - - + + + - - - + + +
TSC2 KO MEFs+empty virus
TSC2 KO MEFs+ WT virus
TSC2 KO MEFs + SE virus
TSC2 KO MEFs + SA virus
Insulin
- - - + + + - - - + + + - - - + + + - - - + + +
AKT
TSC2 KO MEFs+empty virus
TSC2 KO MEFs+ WT virus
TSC2 KO MEFs + SE virus
TSC2 KO MEFs + SA virus
- - - + + + - - - + + + - - - + + + - - - + + +
Insulin
TSC2
260
160

Figure 2B

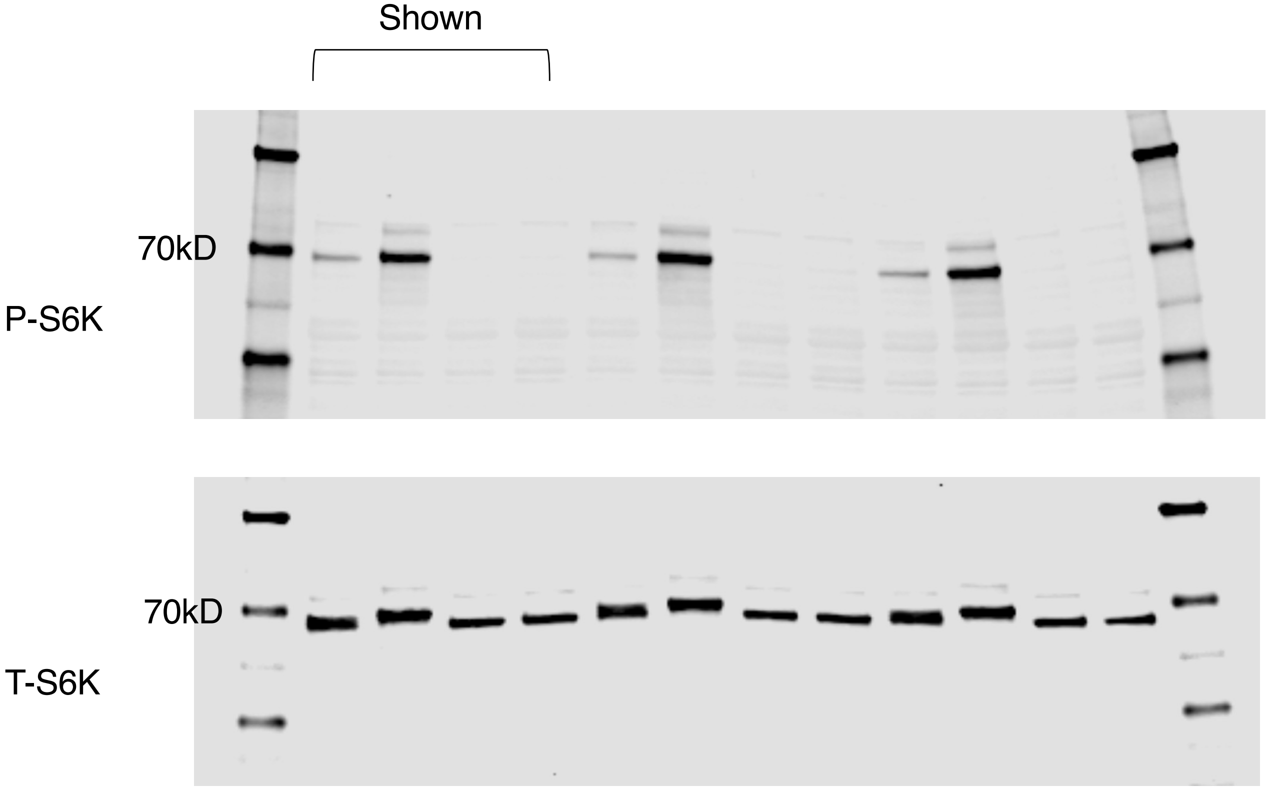

Lanes 1, 5, 9 : Control vehicle
Lanes 2, 6, 10: Insulin
Lanes 3, 7, 11: Insulin + Rapamycin
Lanes 4, 8, 12: Insulin + Torkinib

Figure 3B

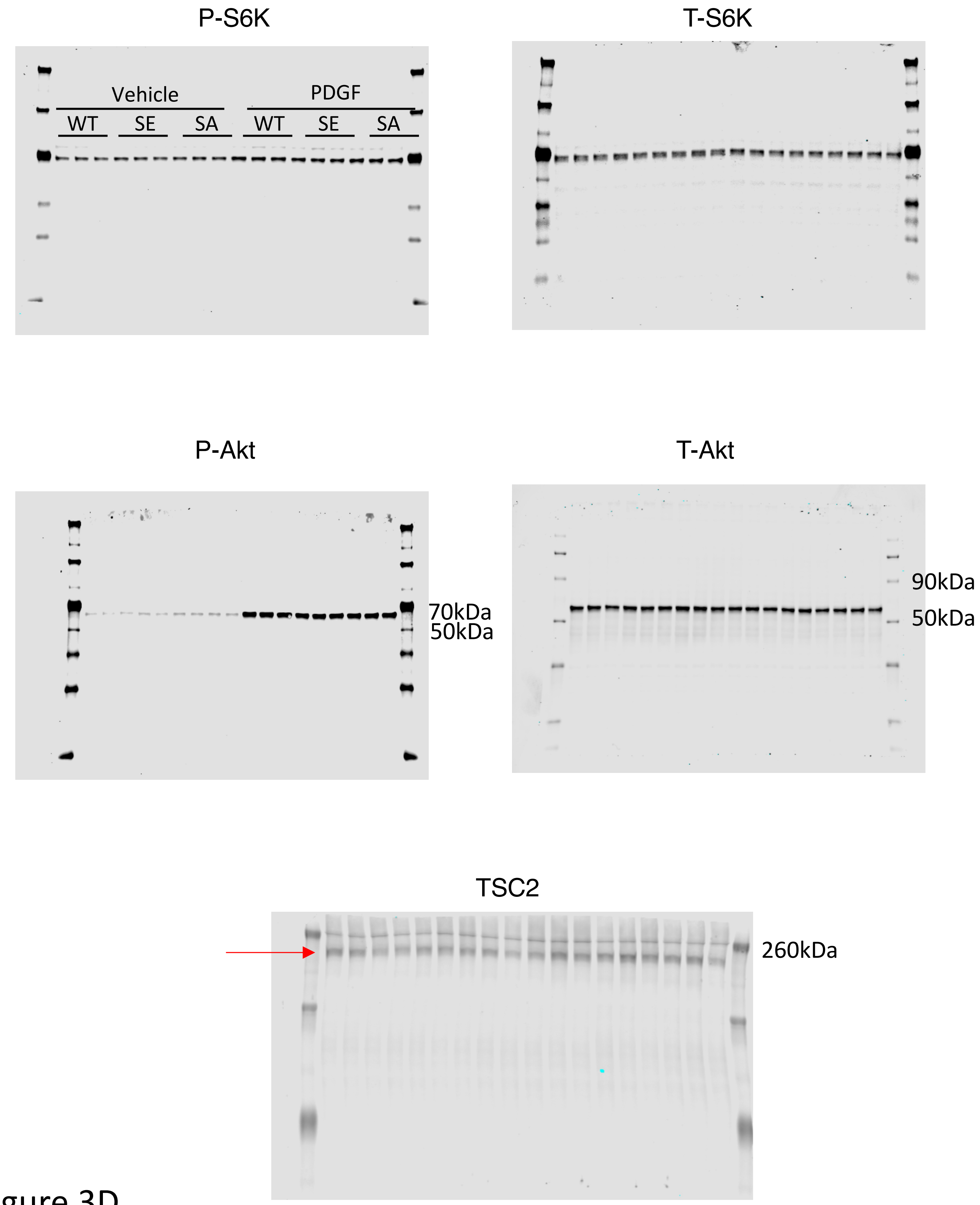
P-S6K
Vehicle
PDGF
WT
SE
SA
WT
SE
SA
T-S6K
P-Akt
70kDa
50kDa
T-Akt
90kDa
50kDa
TSC2
260kDa

Figure 3D
