# Supplementary material for: Single serine on TSC2 exerts biased control over mTORC1 activation mediated by ERK1/2 but not Akt
Source: Life Sci Alliance. 2022 Mar 14;5(6):e202101169. doi: 10.26508/lsa.202101169 (PMC8921838; doi:10.26508/lsa.202101169)
Supplement: Supplementary file 7 [file LSA-2021-01169_SdataF4.1.pdf]

Lanes Used: 12-17: KO MEFs transfected with WT TSC2. Data shown in Fig 4A are from lanes 12-17: 12: Vehicle, 13: ET-1 10 nM; 14: ET1 100 nM; 15: Thrombin 1 nM; 16: Thrombin 10 nM, 17: Thrombin 100 nM.

pERK1/2

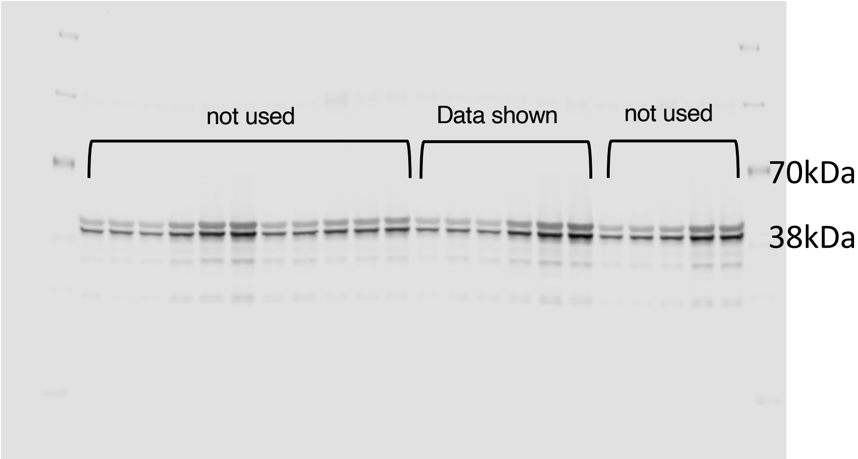

P-Akt

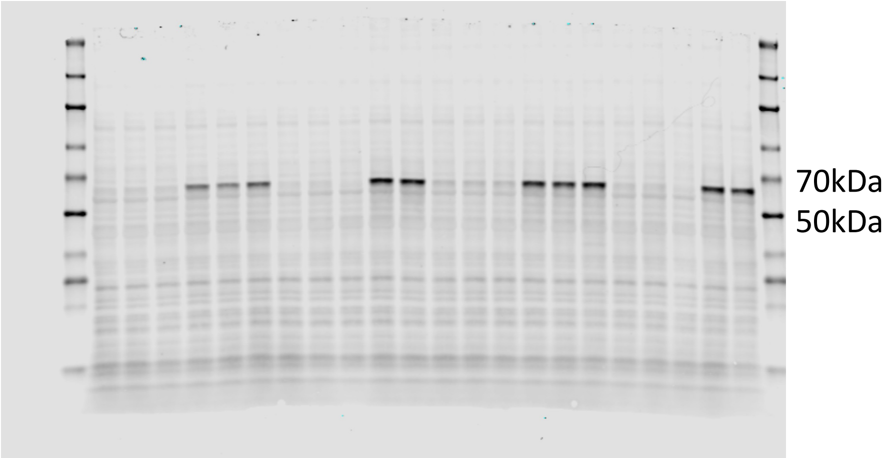

Total Protein

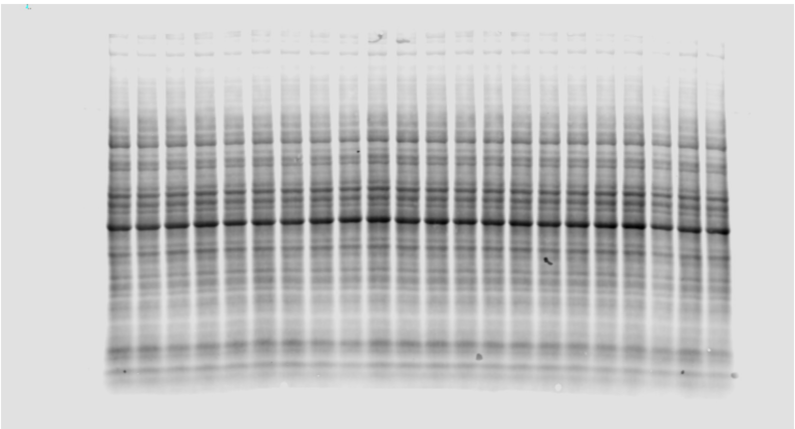

Figure 4A

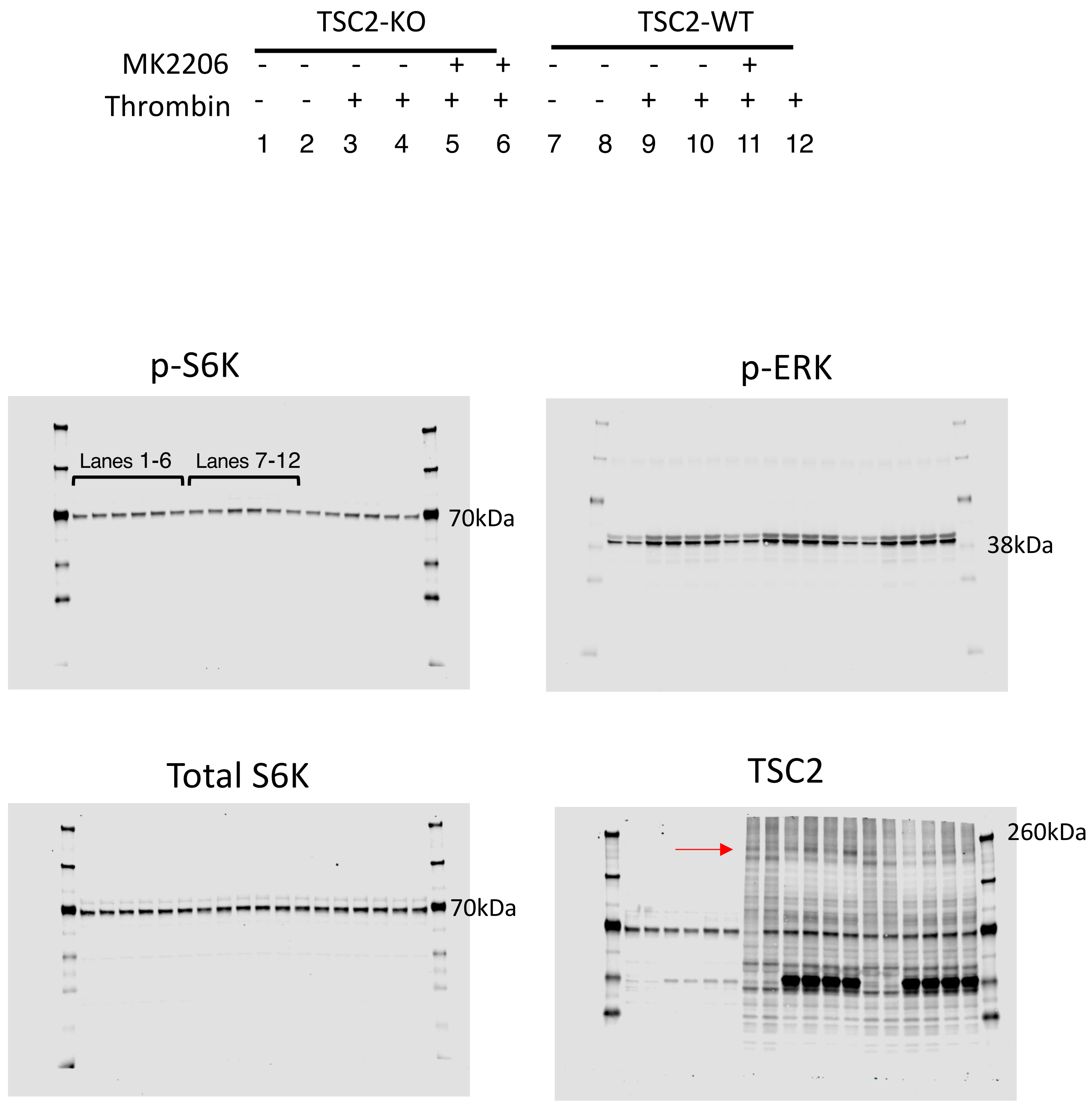
TSC2-KO
TSC2-WT
MK2206 - - - - + + - - - - +
Thrombin - - + + + + - - + + + +
1 2 3 4 5 6 7 8 9 10 11 12
p-S6K
Lanes 1-6
Lanes 7-12
70kDa
p-ERK
38kDa
Total S6K
70kDa
TSC2
260kDa

Figure 4B

Lanes 7-12 shown in Figure 4C

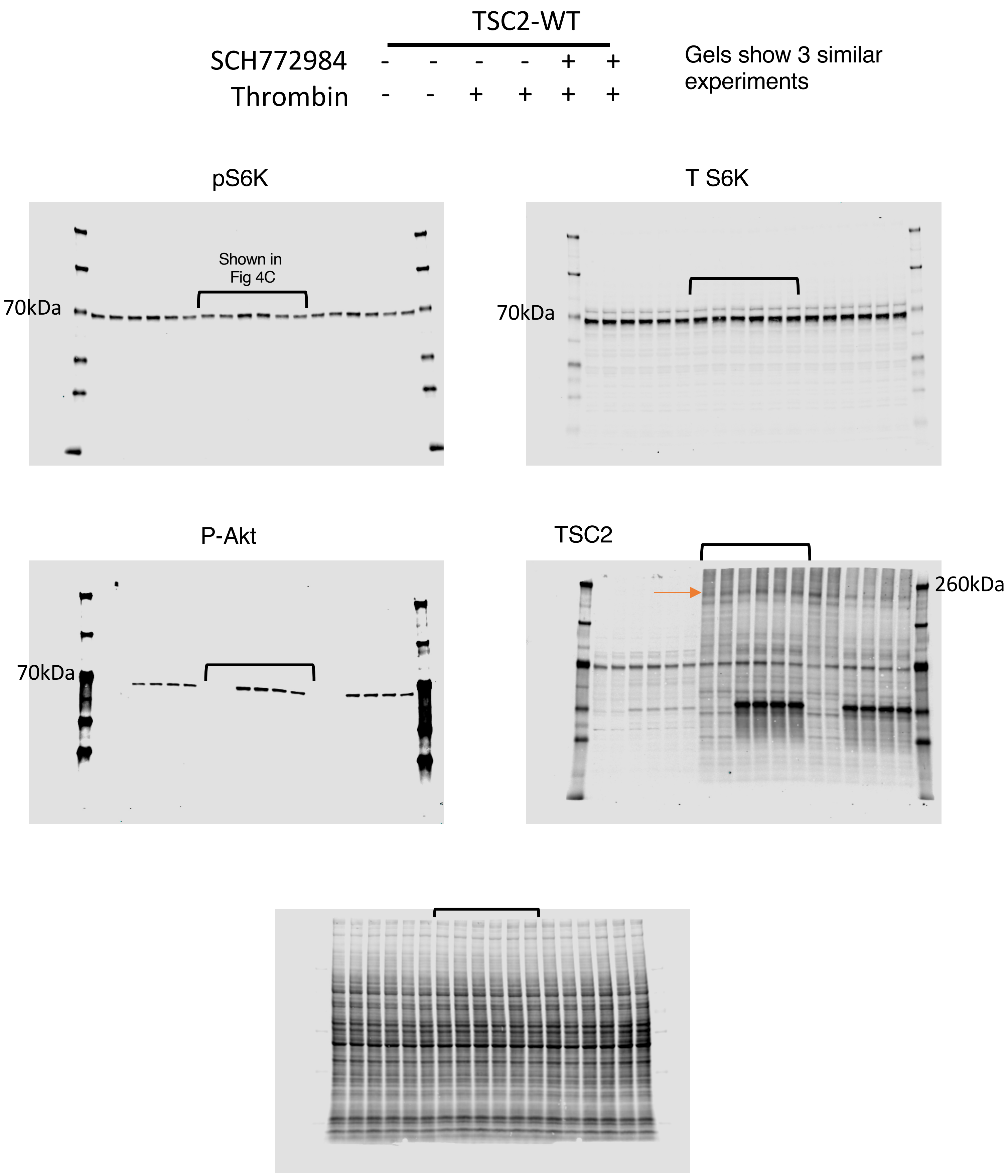

Figure 4C

Lanes 1-3. WT TSC2+ Vehicle
4-6 WT TSC2 + Thrombin
7-9 WT TSC2 + Thrombin + MK2206

10-12 SE TSC2 + Vehicle
13-15 SE TSC2+ Thrombin
16-18 SE TSC2+Thrombin+MK2206

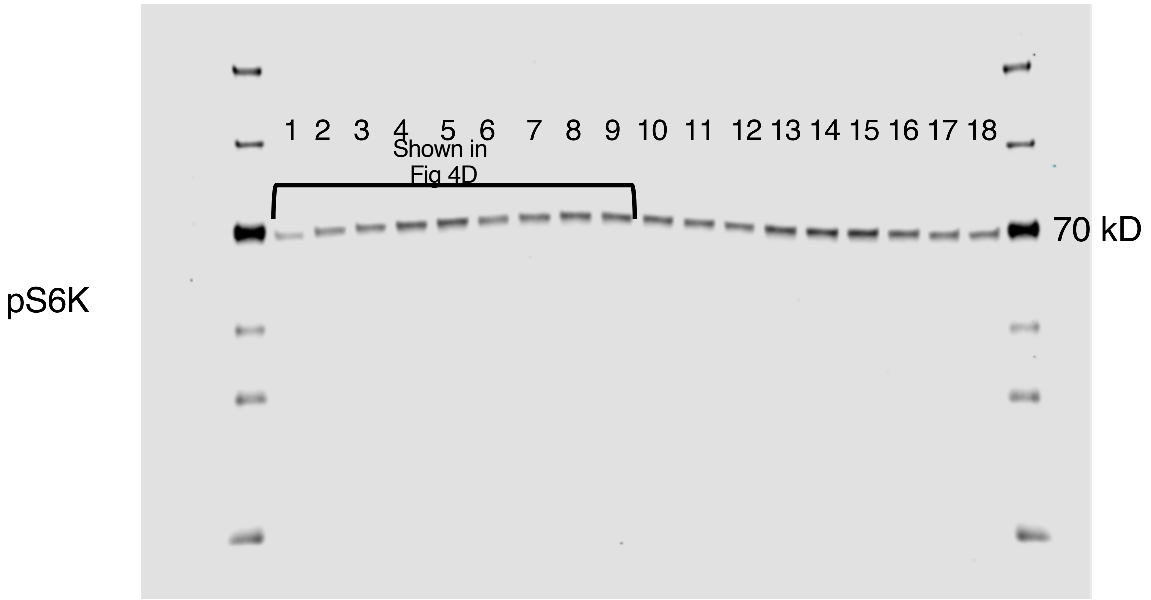

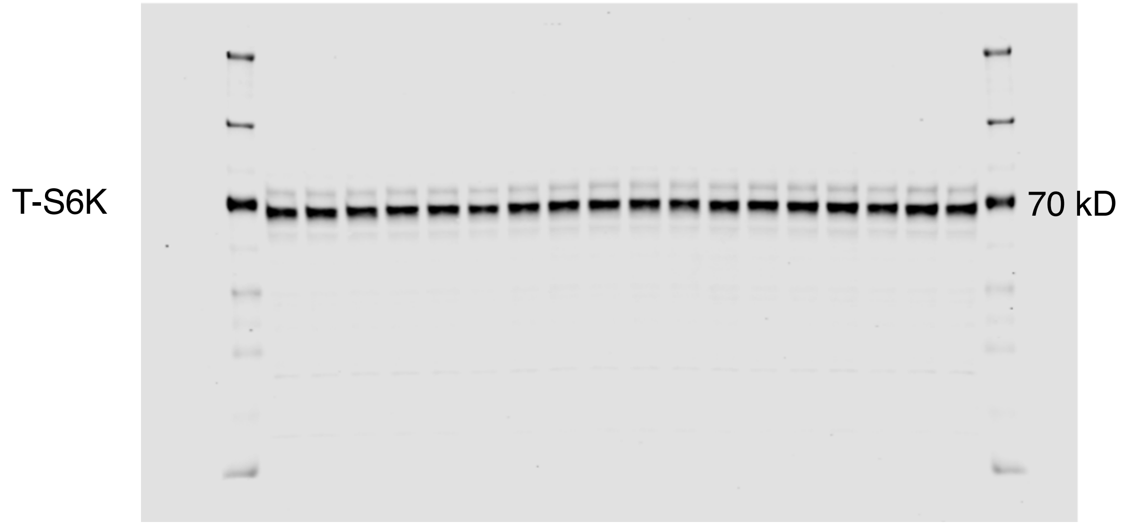

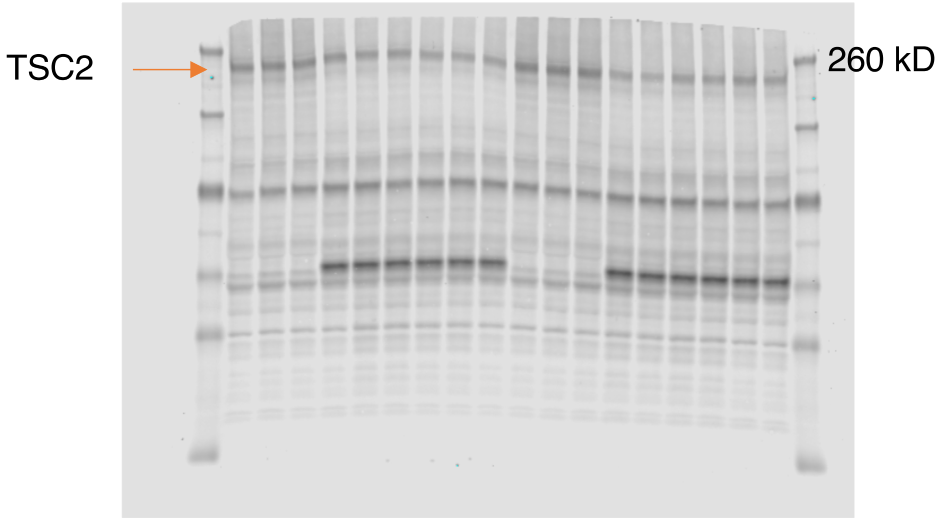

Figure 4D

pS1364 TSC2

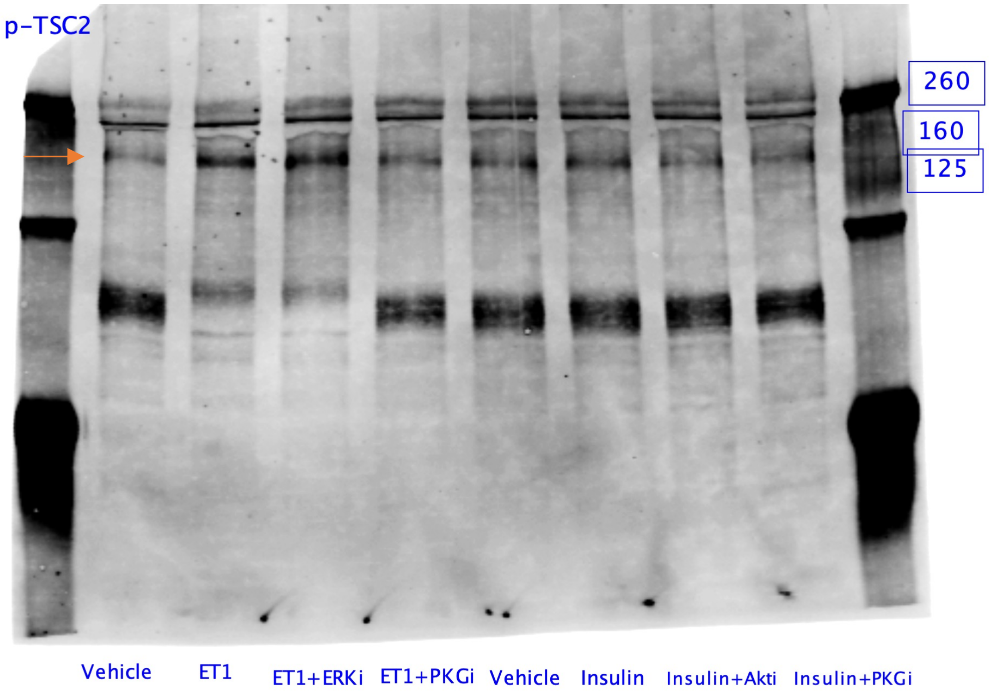

Total Protein

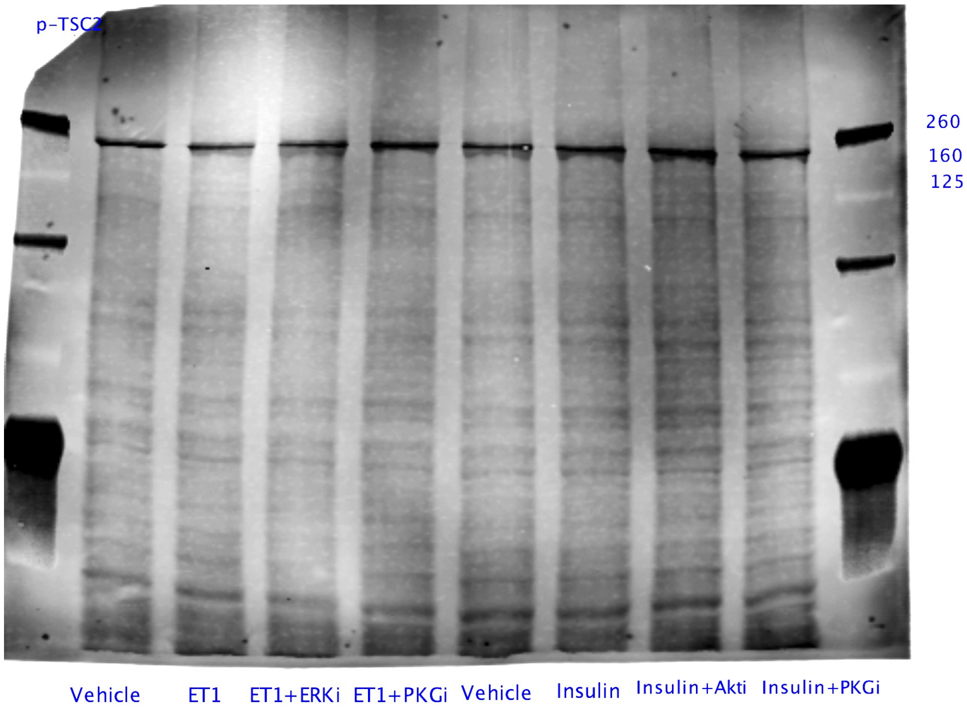

Figure 4H
