# Supplementary figures and images for: Single serine on TSC2 exerts biased control over mTORC1 activation mediated by ERK1/2 but not Akt
Source: Life Sci Alliance. 2022 Mar 14;5(6):e202101169. doi: 10.26508/lsa.202101169 (PMC8921838; doi:10.26508/lsa.202101169)

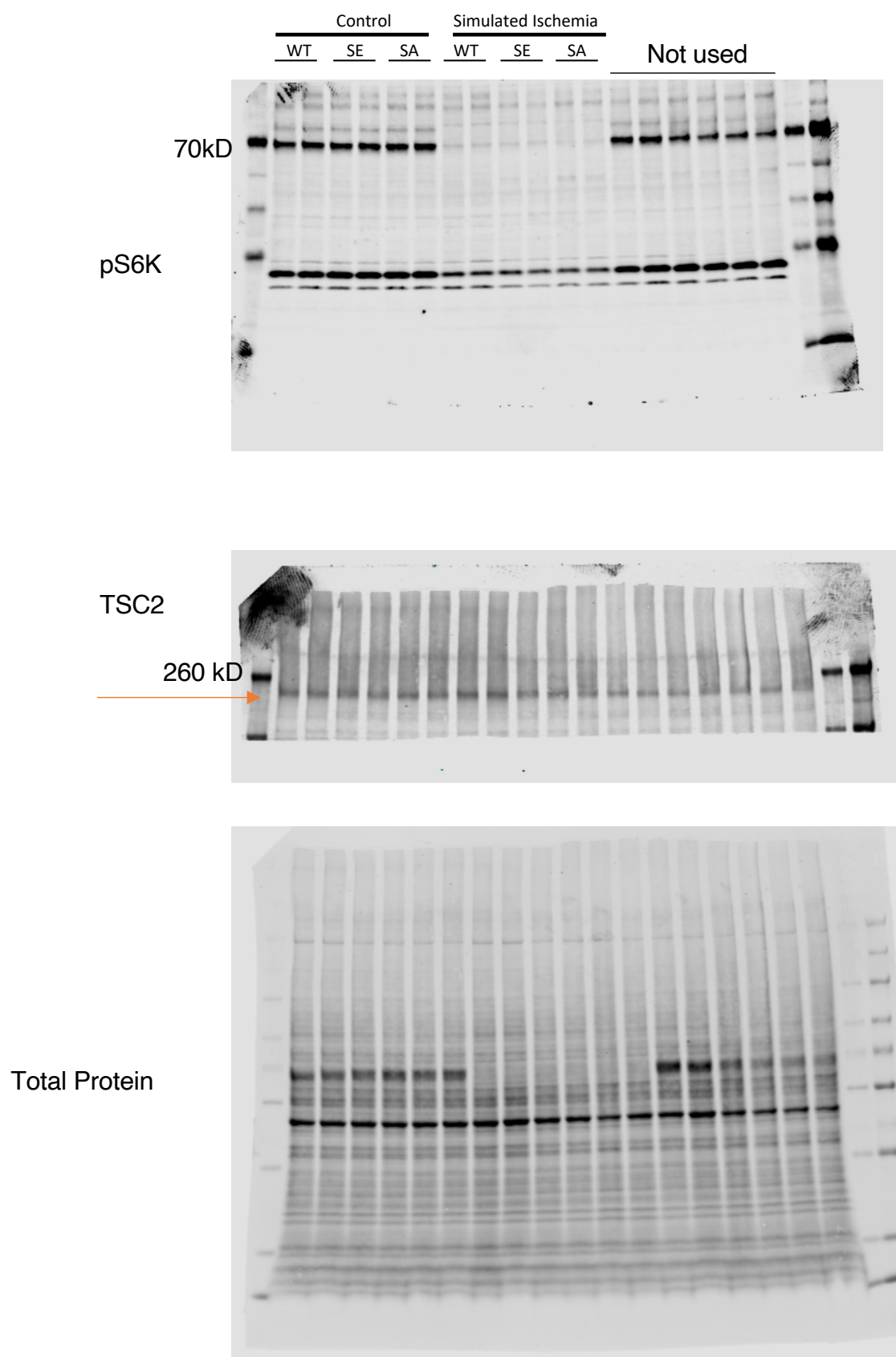

Figure 5A Raw Gels

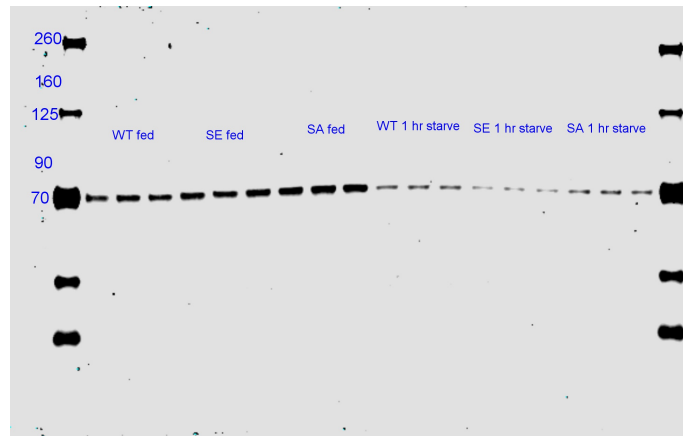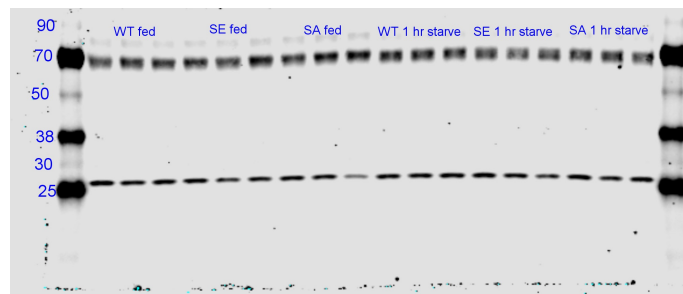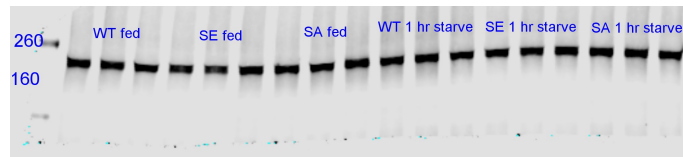

Figure 5B Raw Gels

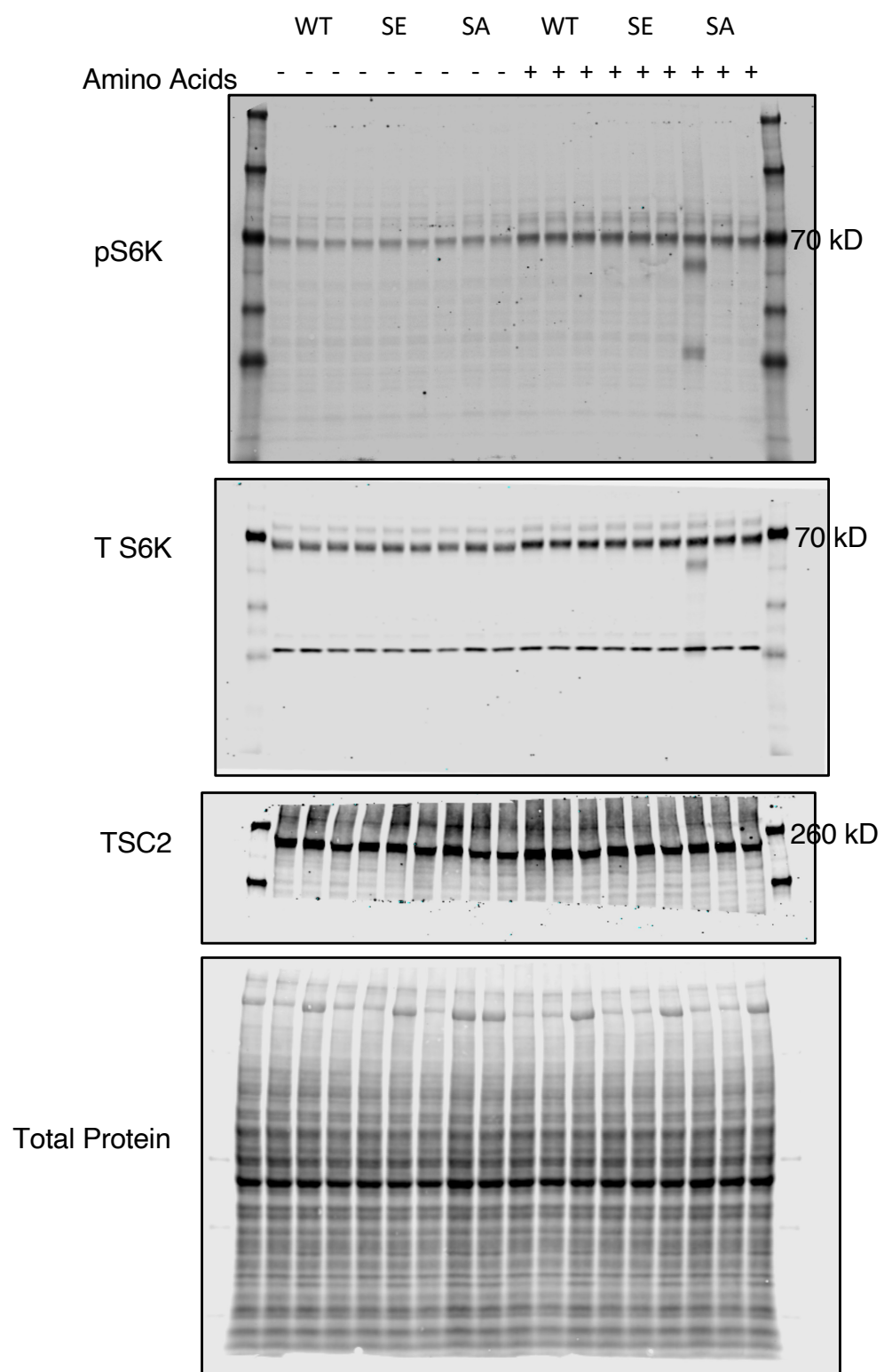

Figure 5C Raw Gels

Supplement: Supplementary file 9 [file LSA-2021-01169_SdataF5.1.pdf]
